# Inhibition of Hedgehog signaling ameliorates foam cell formation by promoting autophagy in early atherosclerosis

**DOI:** 10.1038/s41419-023-06270-5

**Published:** 2023-11-14

**Authors:** Yuting Zhang, Weijuan Xin, Xiaozhi Hu, Hanqi Wang, Xiaomiao Ye, Caili Xu, Yanyang Nan, Zhengyu Wu, Dianwen Ju, Jiajun Fan

**Affiliations:** 1 Department of Biological Medicines & Shanghai Engineering Research Center of Immunotherapeutics, Fudan University School of Pharmacy, Shanghai, China; 2 Department of Gynecology, Obstetrics and Gynecology Hospital of Fudan University, Shanghai, 200090 China; 3 Department of Cardiology, Minhang Hospital, Fudan University, 170 Xinsong Road, Shanghai, 201199 China; 4 TAU Cambridge Ltd, The Bradfield Centre UNIT 184, Cambridge Science Park, CB4 0GA Cambridge, UK; 5 Fudan Zhangjiang Institute, Shanghai, 201203 China

**Keywords:** Mechanisms of disease, Valvular disease

## Abstract

Macrophages are the origin of most foam cells in the early stage of atherosclerotic plaques. However, the mechanism involved in the formation of macrophage-derived foam cell formation remains unclear. Here, we revealed that the hedgehog (Hh) signaling is critical in autophagy-lysosome pathway regulation and macrophage-derived foam cell formation. Inhibition of Hh signaling by vismodegib ameliorated lipid deposition and oxidative stress level in atherosclerotic plaques in high-fat diet-fed apoE^−/−^ mice. For mechanistic study, how the Hh signaling modulate the process of foam cell formation were accessed afterward. Unexpectedly, we found that suppression of Hh signaling in apoE^−/−^ mice had no significant impact on circulating cholesterol levels, indicating that Hh pathway modulate the procession of atherosclerotic plaque not through a traditional lipid-lowing mechanism. Instead, vismodegib was found to accelerate autophagosomes maturation as well as cholesterol efflux in macrophage-derived foam cell and in turn improve foam cell formation, while autophagy inhibitors (LY294002 or CQ) administration significantly attenuated vismodegib-induced cholesterol efflux and reversed the effect on foam cell formation. Therefore, our result demonstrated that inhibition of the Hh signaling pathway increases cholesterol efflux and ameliorates macrophage-derived foam cell formation by promoting autophagy in vitro. Our data thus suggested a novel therapeutic target of atherosclerosis and indicated the potential of vismodegib to treat atherosclerosis.

## Introduction

Atherosclerosis is a metabolic and chronic inflammatory vascular disorder that can be accompanied by lipid accumulation as well as abnormal cell migration and proliferation under the endothelium, leading to life-threatening acute cardiovascular diseases [1, 2]. In the early stage of atherosclerosis, circulating monocytes are recruited into the sub-endothelium and differentiate into macrophages and then become foam cells by internalizing lipid particles [3, 4]. The traditional care for atherosclerotic patients is to help them maintain their lipid and cholesterol levels in the circulation with statins or other lipid lowing agents. Nevertheless, over 50% of patients barely respond, and even experience adverse effects such as myopathy, rhabdomyolysis and some renal side-effects in statins treatment [5–7]. Thus, it is urgent to find innovative therapeutic targets for the treatment of atherosclerosis.

Hedgehog (Hh) signaling pathway is widely expressed in various cells with diverse important functions involved in embryonic development, tissue maintenance, stem cell development and differentiation [8, 9]. Activation of Hh signaling pathway is triggered by Hh family proteins, which bind to Patched-1 (PTCH1) protein located on the cell membrane. Conjunction of Hh proteins and PTCH1 releases Smoothen (SMO) and triggers downstream signaling activation which in turn activates GLI transcription factors, and eventually finish the regulation of signaling [8, 10, 11]. Hh pathway has been proved to play an essential role in promoting cardiovascular system maturation including yolk sac vascularization, early vascular and heart development during embryonic development [12–14]. In adults, increased ligands for Hh family proteins, particularly sonic Hedgehog in plasma, facilitates revascularization in ischemic tissues including myocardial ischemia reperfusion injury [15–17]. Recently, a bunch of evidence has been raised to indicate a correlation between atherosclerosis and Hh signaling. PTCH1 has been reported to overexpressed in human advanced atherosclerotic lesions, especially in CD68 positive macrophages [18], while in the atherosclerotic plaques from apoE^−/−^ and LDLR^−/−^ mice with high-fat diet, HHIPL1, which plays a positive role in Hh signaling pathway activation, is overexpressed in VSMCs of plaque and accelerates atherogenesis in the early stage of atherosclerosis [19]. However, L Beckers et al. showed that inhibition of Hh signaling with an antibody to three Hh ligands slightly aggravates advanced atherosclerosis [20]. Thus, how the Hh signaling pathway modulate atherosclerosis still remains controversial.

Autophagy, as a conserved mechanism, transports abnormal protein, damaged organelles or some other materials to the lysosome where cytoplasmic materials are degraded into nutrients for reuse [21, 22]. Generally, abnormal increase of intracellular lipid content upregulates autophagy which facilitates lipid metabolism by promoting lipid breakdown [23, 24]. However, damaged autophagy in atherosclerotic lesions, as a consequence of over-loaded lipid, contributes to lipotoxicity which further aggravates autophagy impairment and mitochondrial injury, thereby inducing inflammation and cell death and following exacerbated atherosclerotic plaque [25–27]. Evidence showed that activated autophagy in foam cells promotes cholesterol efflux, which ameliorates foam cell formation and atherosclerotic burden [28, 29]. In foam cells, autophagosome packaged-lipid droplets (LDs) are trafficked to lysosomes where free cholesterols originating from hydrolyzation of LDs by lysosomal acid lipase are generated for cholesterol efflux [30–32]. According to the results of previous studies, activation of Hh pathway induces the inhibition of autophagy in different cell lines including endothelial cells [11], endometrial cells [33], lung cells [34], colon cancer cells [35] and non-small cell lung cancer cells [36]. For this reason, we formulated a hypothesis that the activation of Hh signaling pathway in atherosclerosis might have an impact on autophagy, which subsequentially influenced lipid accumulation in foam cells.

In our study, we found Hh signaling pathway was activated, which thereby promoted atherosclerosis in the early stage, and exacerbated atherosclerosis. We further demonstrated the protective effects of vismodegib against atherosclerosis, including reduction in lipid deposition, plaque area and ROS level, suggesting Hh signaling as an innovative, potential therapeutic target for the treatment of atherosclerosis. Moreover, our results revealed for the first time the relationship between Hh signaling pathway and autophagy-dependent lipophagy in macrophage-derived foam cells.

## Results

### Activation of Hh signaling pathway promoted lipid deposition and oxidized stress level of foam cells

**Fig. 1:**
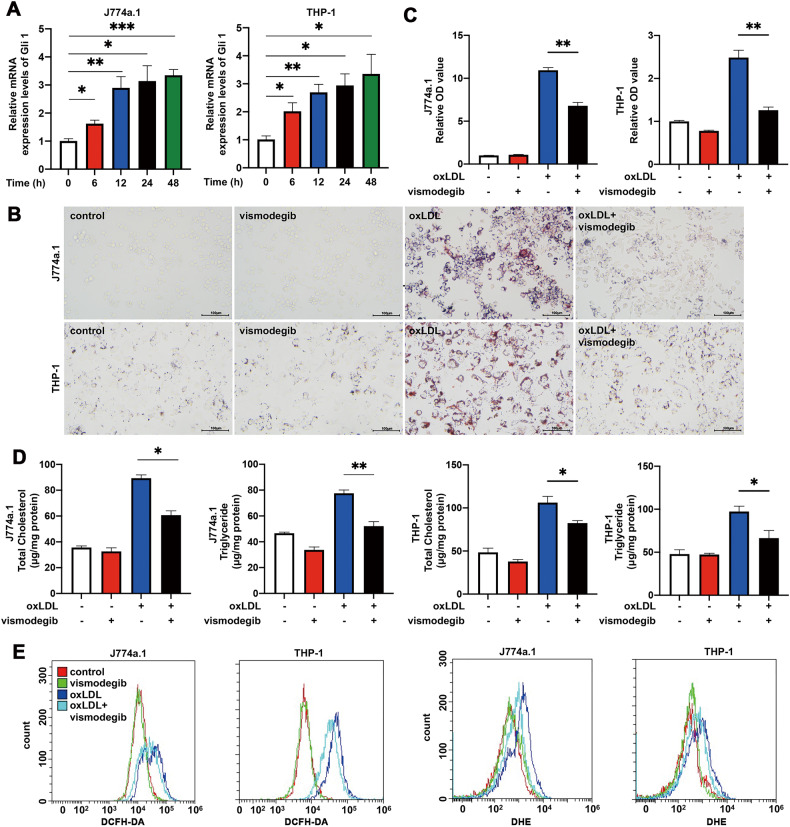
Hh signaling is activated in macrophage-derived foam cells. **A** The relative expression of Gli1 in J774a.1 macrophages and THP-1 were treated with oxLDL at different time points (*n* = 4). **B**, **C** Images of ORO-stained macrophages. J774a.1 and THP-1 cells were stimulated with vehicle control, Hh signaling inhibitor (vismodegib), oxLDL, or vismodegib and oxLDL for 24 h then stained for observing. **C** The ORO OD values were measured at 510 nm of each group in (**B**) (*n* = 3). Total cholesterol (**D**) or Triglyceride (**E**) levels in macrophages described in (**B**) (*n* = 3). **F** The histogram of DCFH-DA and DHE fluorescence of macrophages described in (**B**). *p < 0.05, **p < 0.01, ***p < 0.001.

Canonical Hh signaling activation increases GLI family zinc finger 1 (*Gli1*) gene expression, which is one of critical pathway members. To evaluate the impacts of atherosclerotic stimuli on Hh signaling, J774a.1 mice macrophages and THP-1 cells were incubated with oxLDL to construct foam cell. As shown in Fig. 1A, treatment of oxLDL remarkably increased Gli1 expression in time-dependent manner, suggesting that Hh signaling activated during the process of macrophage transforming into foam cells.

We further investigated whether Hh signaling pathway involved in the process of foam cell formation. J774a.1 and THP-1 macrophages were stimulated with oxLDL and /or vismodegib for 24 h. As shown in Fig. 1B, C, Oil Red O analysis indicated that inhibiting Hh signaling pathway by vismodegib resulted in a remarkable decrease in lipid deposition when compared to cells treated with oxLDL only. Similarly, intracellular TC and TG contents of those two cell lines cultured with oxLDL for 24 h were significantly increased in comparison with the control, which could be attenuated by vismodegib (Fig. 1D). These results suggested that inhibition of Hh signaling alleviated lipid deposition in macrophages.

Furthermore, we challenged whether Hh pathway could trigger foam cell formation in vascular smooth cells (VSMCs), one of the most important cells that involved in the progression of advanced atherosclerosis. After oxLDL treatment, Gli1 expression increased in a time-dependent manner in mouse VSMCs (Fig. S1A), while suppression of Hh pathway by vismodegib could alleviated lipid accumulation in VSMCs, with decreased TC and TG contents in cells (Fig. S1B–D), indicating that inhibition of Hh signaling ameliorated lipid deposition and foam cell formation in VSMCs.

As was reported previously, overloaded lipid could trigger reactive oxygen species (ROS) as well as lipid peroxidation which contribute to oxidative stress in foam cells. Thus, we also challenged whether vismodegib treatment could attenuate the oxidative stress level in foam cells. DCFH-DA and DHE probe were applied to detect ROS and lipid peroxides, respectively. Compared with cells stimulated with oxLDL only, vismodegib treatment significantly decreased oxidative stress level in foam cells (Fig. 1E), indicating that Hh suppression could attenuate oxidative stress in foam cells.

Thus, our results demonstrated the activated Hh signaling pathway in oxLDL-treated macrophages and VSMCs, which was involved in lipid deposition, oxidative stress-related injury and foam cell formation of those cells.

### Inhibition of Hh signaling pathway alleviates atherosclerosis in vivo

**Fig. 2:**
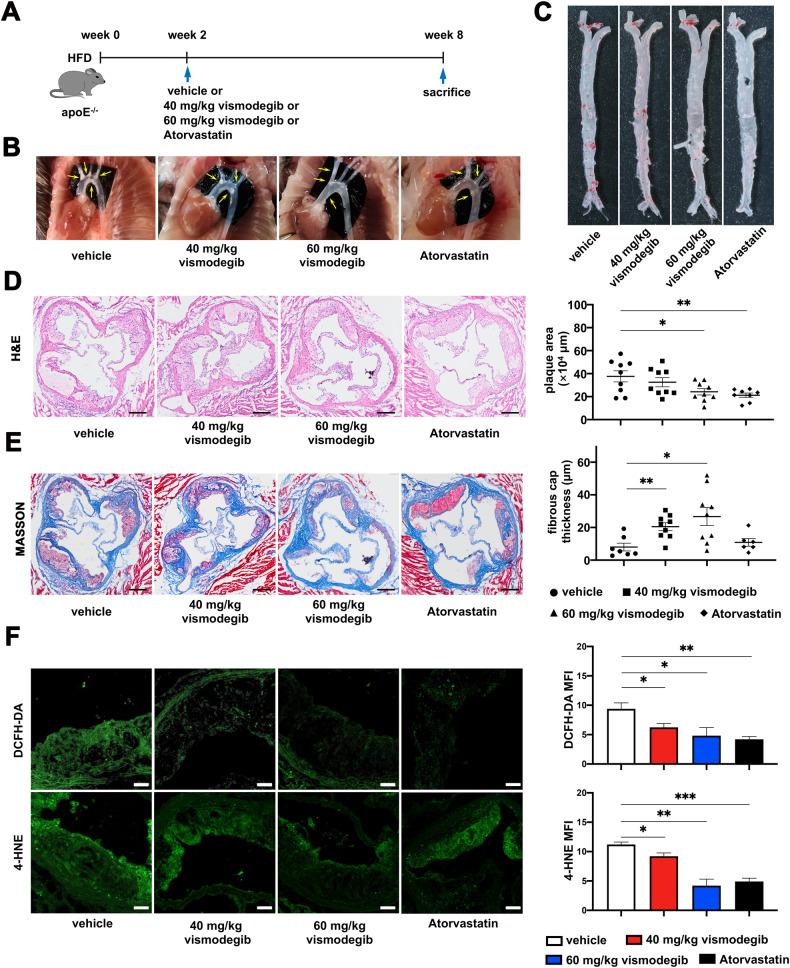
Inhibition of Hh signaling pathway ameliorates atherosclerosis and oxidative level in HFD-fed apoE^-/-^ mice. **A** Experimental schema. ApoE^−/−^ mice were fed with HFD for 2 weeks and administered with vehicle control, 40 mg/kg vismodegib, 60 mg/kg vismodegib or atorvastatin for another 6 weeks. **B** The representative images of plaques in the aortic arch (yellow arrows) of apoE^−/−^ mice in each group. **C** ORO staining of *en face* aorta obtained from apoE^−/−^ mice of each group. **D** Images of H&E staining of aortic sinus cross-sections of apoE^−/−^ mice in each group. Plaque area in groups described in A was measured according to the results of H&E staining (*n* = 8 ~ 9). **E** Images of MASSON staining of aortic sinus cross-sections of apoE^−/−^ mice of apoE^−/−^ mice in each group. Fibrous cap thickness of aortic sinus in groups were measured (*n* = 8 ~ 9). **F** Images of DCFH-DA staining and 4-HNE immunofluorescent staining of aortic sinus cross-sections of apoE^−/−^ mice in groups. MFI in the plaque of aortic sinus in groups were calculated (*n* = 4). Scale bar = 200 μm (**D**, **E**) Scale bar = 50 μm (**F**) *p < 0.05, **p < 0.01, ***p < 0.001.

To figure out the role of Hh pathway in the development of atherosclerosis, apoE^−/−^ mice were fed with a high-fat diet (HFD) for 2 weeks and then maintained on HFD for additional 6 weeks with vismodegib at 40 or 60 mg/kg (Fig. 2A). Similar with the mice treated with atorvastatin, the positive controls, mice treated with vismodegib developed fewer lesions in the whole aorta when compared to that of the vehicles (Fig. 2B, C). Besides, we also found that vismodegib treatment significantly decreased plaque area in aortic root (Fig. 2D). Next, we further challenged whether vismodegib could contribute to the stability of the plaque. Masson staining was employed to determine the collagen content as well as the fibrous-cap thickness of the plaques. Our results revealed that vismodegib treatment had no prominent effect on collagen content but the thickness of fibrous cap (Fig. 2E), which indicated that plaque stability improved after vismodegib treatment. ROS are reactive molecules and free radicals which derived from molecular oxygen indicating oxidative stress level in tissue. In atherosclerotic plaque, ROS level were related in cell stress, apoptosis and inflammation, which aggravated vulnerability of plaque. Thus, we labeled ROS in plaques by DCFH-DA probe to characterize oxidative stress level of atherosclerosis in vivo. We further used 4-HNE adducts to indicate oxidative damage. As shown in Fig. 2F, ROS and lipid peroxidation level both dramatically decreased in apoE^-/-^ mice treated with vismodegib or atorvastatin, indicating a lower oxidative stress level in vismodegib treatment.

Therefore, our results demonstrated that inhibition of Hh pathway by vismodegib decreased plaque area, improved plaque vulnerability, reduced oxidative stress level and postponed the atherosclerotic progression.

### Inhibition of Hedgehog signaling pathway reduced lipid content in mouse atherosclerotic plaques without circular lipid level

**Fig. 3:**
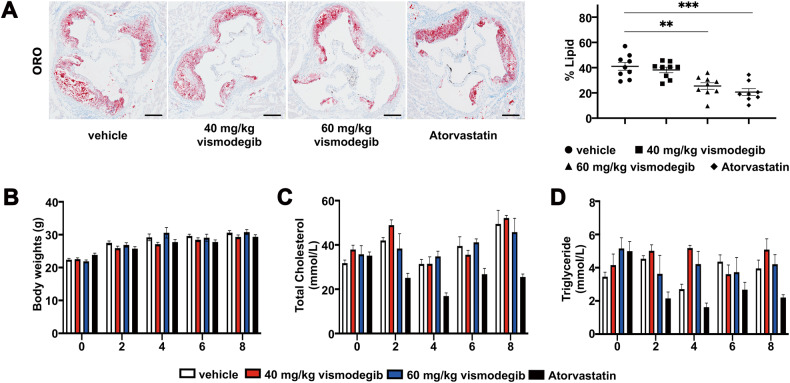
Inhibition of Hh signaling pathway reduces lipid content in atherosclerotic plaques without changing circulated lipid level. **A** Images of ORO staining of aortic sinus cross-sections of apoE^−/−^ mice in each group. Percentage of lipid content in the plaque of aortic sinus in groups described in (**A**) (*n* = 8 ~ 9). Scale bar = 200 μm. **B**–**D** The body weights, Total cholesterol and Triglyceride levels in indicated groups of mice at weeks 0, 2, 4, 6, and 8 (*n* = 4 ~ 7). *p < 0.05, **p < 0.01, ***p < 0.001.

To accessed the reason why vismodegib could reduce aortic sinus lesions and vulnerability in vivo, we measured the composition of plaque in different groups of apoE^−/−^ mice. Compared with the vehicles, lipid content of the aortic sinus was significantly reduced in the high-dose vismodegib treatment group (Fig. 3A), indicating that decreased lipid deposition in aortic sinus might be the probable reason that caused the decelerated plaque formation and attenuated aortic arch lesions.

As has been observed in lesions after apoE^−/−^ mice were treated with vismodegib, we wondered whether modulation of lipids was likely to be a probable. The circulating lipid level in plasmas of mice were measured at first. Unexpectedly, serum total cholesterol and triglyceride in plasmas of vismodegib-treated mice revealed no difference compared with the vehicle (Fig. 3B–D), which indicated that Hh pathway modulate the procession of atherosclerotic plaque not through a traditional lipid lowing mechanism.

### Inhibiting Hh signaling pathway ameliorated macrophage-derived foam cell formation by promoting cholesterol efflux

**Fig. 4:**
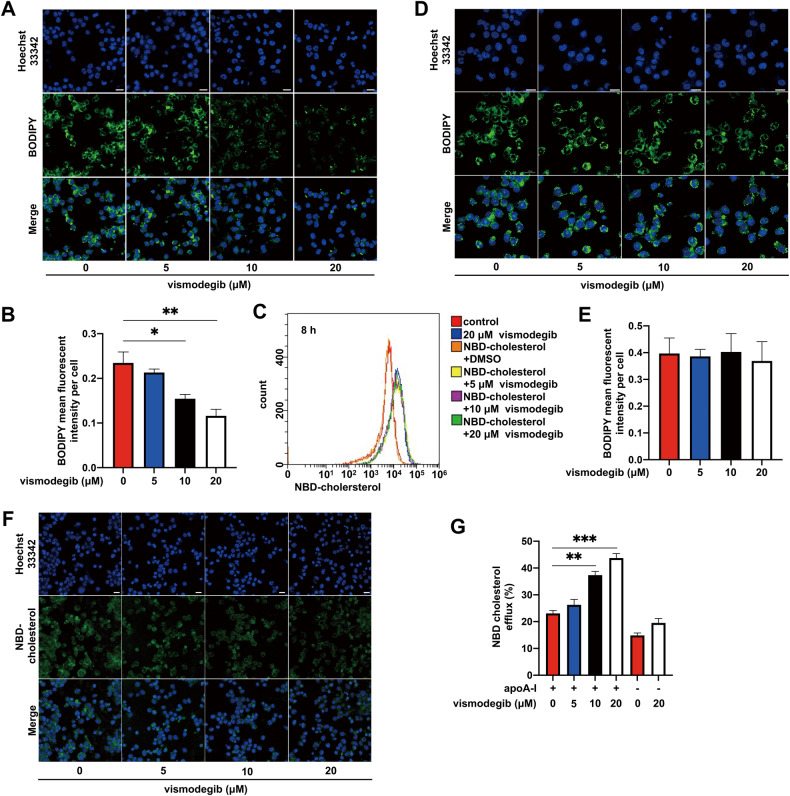
Effects of Hh pathway inhibition on lipid internalization and cholesterol efflux in macrophages. **A** Images of BODIPY staining of macrophages. J774a.1 cells were treated with oxLDL and vismodegib at a concentration of 0, 5, 10 or 20 μM. **B** Per cell BODIPY mean fluorescent intensity of cells described in (**A**) (*n* = 3). **C** The histogram of NBD-cholesterol fluorescence of macrophages. 0, 5, 10 or 20 μM vismodegib pre-treated J774a.1 cells were incubated with NBD-cholesterol for 8 h. **D** Images of BODIPY staining of macrophages. 0, 5, 10 or 20 μM vismodegib pre-treated J774a.1 cells were incubated with oxLDL for 8 h. **E** Per cell BODIPY mean fluorescent intensity of cells described in (**D**) (*n* = 4 or 5). **F** Fluorescent images of 0, 5, 10 or 20 μM vismodegib pre-treated macrophages loaded with NBD-cholesterol after incubated with apoA-I for 1 h. **G** percentage of apoA-I mediated cholesterol efflux of cells described in (**F**) was calculated (*n* = 3). Scale bar = 20 μm. *p < 0.05, **p < 0.01, ***p < 0.001.

In the early stage of atherosclerosis, macrophages, as the main infiltrating cell differentiate into foam cells by uptake of lipoproteins and enormously contribute to atherogenesis. To investigate whether Hh signaling inhibition influence lipid internalization and foam cell formation of macrophages, we utilized BODIPY probe to mark neutral lipid drops in vitro. For in vitro study, the efficiency of Hh signaling inhibition and cellular toxicity of vismodegib was evaluated in different concentration. Our data showed that vismodegib could significant inhibit Gli1 expression but had no remarkable impact on cell viability when its concentration was lower than 50 μM and more than 5 μM (Fig. S2). A lower lipid content was observed under fluorescent microscope after vismodegib treatment for 24 h and the area of lipid drops labeled by BODIPY was markedly reduced in a concentration-dependent manner (Fig. 4A, B). To figure out the reason why vismodegib administration decreased lipid droplets content, we first measured lipid uptake capacity of macrophages. After 24 h treatment of vismodegib, J774a.1 cells were incubated with fluorescent cholesterol tracer NBD-cholesterol [22-(N-(7-Nitrobenz-2-oxa-1,3-diazol-4-yl) amino)-23,24-bisnor-5-cholen-3b-ol] and cell fluorescent intensity was detected by flow cytometry after 8 h incubation. As shown in Fig. 4C, we found that phagocytosis of NBD-cholesterol in macrophages was not affected by Hh signaling inhibition as evidenced by flow cytometry. Meanwhile, J774a.1 cells were stimulated with vismodegib for 24 h prior to incubation with ox-LDL as well. Immunofluorescent analysis of BODIPY staining also indicated that the uptake ability of ox-LDL was not influenced by vismodegib (Fig. 4D, E). We next investigated whether vismodegib treatment affects cholesterol efflux which facilitates intracellular lipid content reduction. (NBD-cholesterol)-loaded J774a.1 cells were stimulated with vismodegib and then incubated with lipid-poor apoA-I, a kind of high-density lipoproteins, to evaluate cholesterol efflux. As shown in Fig. 4F, G, vismodegib treatment promoted cholesterol efflux to apoA-I in comparison with control group. Finally, the effect of vismodegib on cellular cholesterol synthesis was also determined. The total cholesterol and precursors (lanosterol lathosterol and desmosterol) of J774a.1 cells were quantified by GC/MS after cells were treated with vismodegib for 24 h. Our results showed that content of cholesterol precursors had no difference after vismodegib treatment (Fig. S3). In conclusion, Hh signaling inhibition reduced lipid contents and numbers of lipid droplets by accelerating cholesterol efflux instead of lipid internalization.

### Autophagy-lysosome pathway was activated by inhibition of Hh signaling pathway in macrophages

**Fig. 5:**
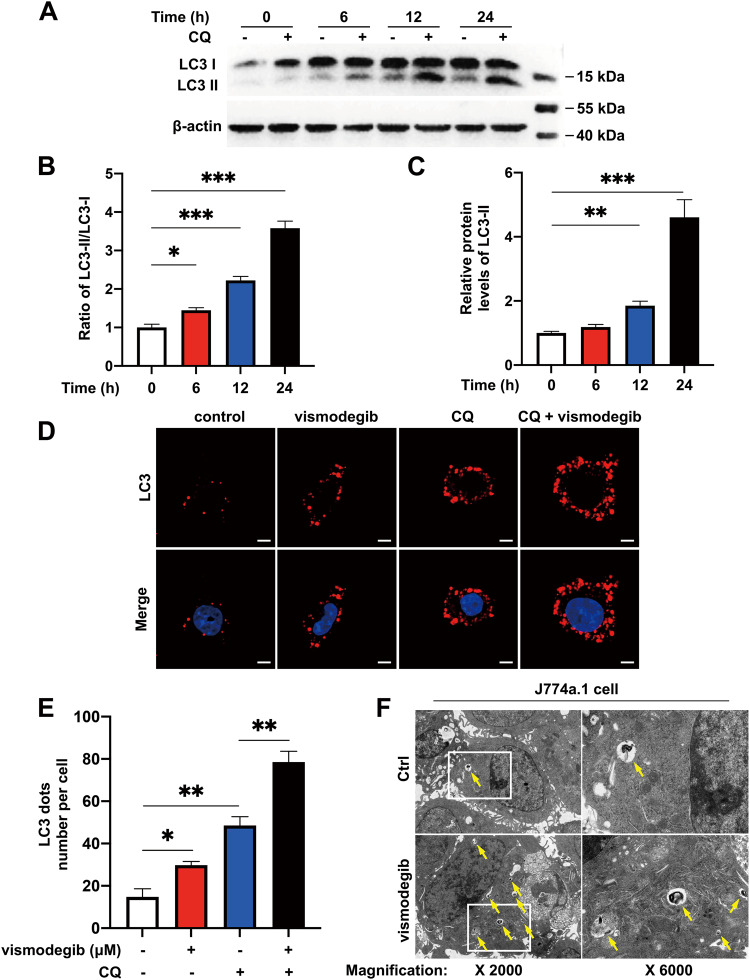
Autophagic flux was activated by Hh signaling inhibition in macrophages. **A** Western blot analysis for LC3-I and LC3-II in J774a.1 cells treated with vismodegib in the presence or absence of CQ at 0, 6, 12 and 24 h. **B**, **C** Ratio of LC3-II/LC3-I or relative protein levels of LC3-II were assessed (*n* = 3) (**D**, **E**) Images of LC3 staining of J774a.1 cells treated with vehicle control, vismodegib, CQ or vismodegib and CQ for 24 h. The number of per cell LC3 dots was calculated (*n* = 4). **F** TEM images of cells treated with or without vismodegib. Autophagosomes were indicated with yellow arrowheads. *p < 0.05, **p < 0.01, ***p < 0.001.

Given the previous reported relationship between autophagy flux and Hh signaling pathway in tumor cells, we postulated that Hh pathway might be participate in autophagy in macrophages. Firstly, we detected the expression of LC3-II protein after inhibition of Hh signaling pathway with vismodegib. As shown in Fig. 5A–C, LC3-II expression augmented in a time-dependent manner, the same is true of the ratio of LC3-II/I. In the process of autophagy, LC3-II participates in autophagosome-lysosome fusion and is broken down with the degradation of autophagolysosome. The accumulation of LC3-II in cytoplasm is probably the consequence of promoted autophagy flux or inhibited autophagolysosome degradation. Accordingly, we used autophagy inhibitor chloroquine (CQ) to block the fusion of autophagosome and lysosome. As shown in Fig. 5D, the combined usage of CQ and vismodegib caused further improvement of LC3-II expression level compared with vismodegib monotreatment over time. Moreover, LC3 protein was stained in vismodegib-treated macrophages in the absence or presence of CQ to indicate autophagic structure. We found that vismodegib treatment increased the number of LC3 dots compared with the control group, and the presence of CQ further augmented LC3 dots number (Fig. 5E, F). These results suggested that vismodegib stimulation had a positive effect on promoting autophagy flux. Additionally, we directly visualized autophagic structure by TEM. As shown in Fig. 5G, exposure to vismodegib promoted the formation of autophagosomes in the cytoplasm of macrophages. Collectively, these data revealed that inhibition of Hh pathway induced autophagy in macrophages.

### Inhibition of Hh pathway promoted cholesterol efflux by inducing autophagy

**Fig. 6:**
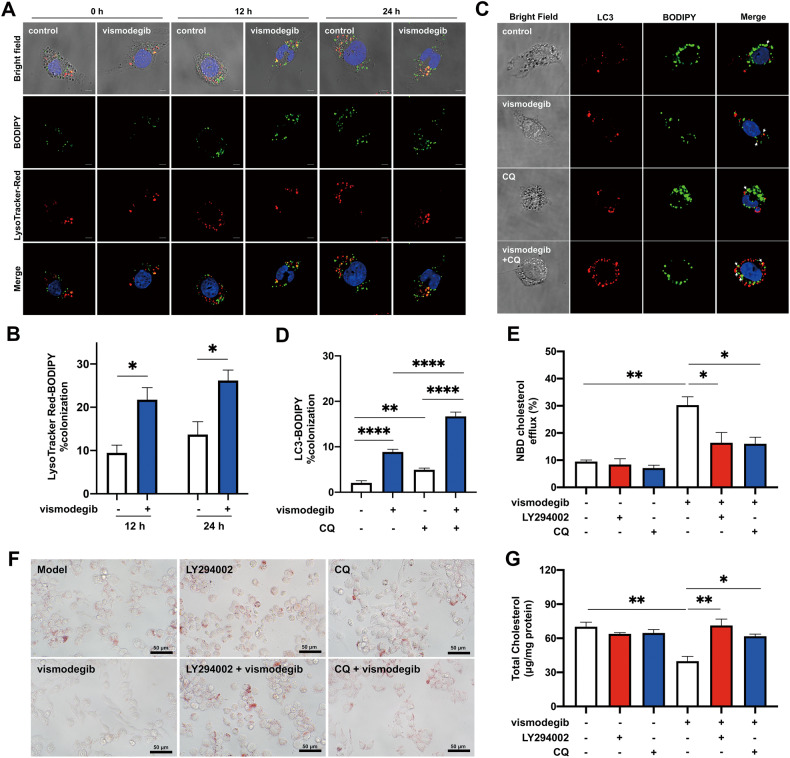
Vismodegib treatment promoted cholesterol efflux and inhibits lipid accumulation by activating autophagy. **A** The images were collected by confocal microscope of LysoTracker Red-BODIPY stained macrophages. OxLDL-loaded J774a.1 cells were treated with vismodegib for 0, 12 or 24 h. Scale bar = 5 μm. **B** The percentage colocalization of LysoTracker Red-BODIPY was calculated (*n* = 3). **C** Confocal images of LC3-BODIPY stained macrophages. OxLDL-loaded J774a.1 cells were treated with vismodegib in the presence or absence of CQ. Scale bar = 5 μm. **D** The percentage colocalization of LC3-BODIPY was assessed (*n* = 4 ~ 7). **E** Cholesterol efflux were measured in vismodegib treated J774a.1 cells with autophagic inhibitor (CQ or LY294002) (*n* = 3). ORO staining images (**F**) or total cholesterol level (**G**) in macrophages described in **E** (*n* = 3). *p < 0.05, **p < 0.01, ***p < 0.001.

Cytoplasmic cholesterol ester (CE) stored in lipid drops of foam cell could be transported to lysosome by autophagosome and hydrolyzed in lysosome full of acid lipase to generate free cholesterol for reverse cholesterol transport. Considering that activation of autophagy in macrophages-derived foam cells has been found to accelerate cholesterol efflux to reduce foam cell formation [28], we speculated that autophagy-lysosome pathway is necessary for cholesterol efflux in vismodegib-treated macrophages. Thus, we first detected whether lysosomes were involved in vismodegib-induced augmentation of cholesterol efflux. As shown in Fig. 6A, B, the colocalization percentage of BODIPY and LysoTracker Red was significantly increased in vismodegib-treated cells compared with controls, suggesting that lysosome-related pathway is activated by inhibition of Hh signaling. Next, we utilized BODIPY to label lipid droplets and LC3 antibody to mark autophagy-related structures in macrophages. Previous studies reported that oxLDL treatment impaired autophagy in macrophages, and autophagy inhibitors 3MA and CQ promoted foam cell formation. As shown in Fig. 6C, D, existence of CQ apparently increased lipid accumulation. Moreover, vismodegib treatment alone enhanced LC3-BODIPY colocalization. When autophagy-lysosome fusion was inhibited with CQ [37], there was an overall increase in the percentage of LC3-BODIPY colocalization compared to untreated cells. And the addition of vismodegib further increased LC3-BODIPY colocalization in CQ-treated cells.

To further figure out whether it is causality between increased autophagy and promoted cholesterol efflux rate and decreased lipid deposition observed in vismodegib treated macrophages, J774a.1 cells were pretreated with LY294002 or CQ to inhibit autophagy. As shown in Fig. 6E, the presence of LY294002 or CQ abolished the promotion of cholesterol efflux rate in vismodegib-treated cells. And the effect of vismodegib in reducing lipid deposition and cholesterol content was also suppressed by LY294002 and CQ (Fig. 6F, G). Collectively, inhibition of Hh pathway by vismodegib activated autophagy, which promoted cholesterol efflux and decreased lipid accumulation, thus ameliorating foam cell formation in macrophages.

## Discussion

Atherosclerosis is the pathophysiological mechanisms underlying various cardiovascular diseases such as stroke and heart attack. Clinical therapeutic strategies for atherosclerosis are mainly focused on maintaining a lower level of blood LDL by statins, NPC1L1 and PCSK9 inhibitors. Nevertheless, the effectiveness of simple lipid-lowing in the treatment of atherosclerosis is not as good as expected. A new and effective therapeutic target of atherosclerotic treatment needed to be found. Here, we demonstrated the inhibitor of Hh signaling pathway, vismodegib, alleviated lipid deposition, lesion area and oxidative stress level in early atherosclerosis through mechanisms independent of serum lipid levels. Vismodegib, an SMO inhibitor, was approved by the United States Food and Drug Administration (FDA) in January 2012 for the treatment of symptomatic metastatic basal cell carcinoma (BBC) and locally advanced BBC in adult patients [38]. In this study, we found vismodegib treatment could decrease lipid deposition in the early stage of atherosclerosis without serum lipid content changing, providing a new potential treatment strategy for atherosclerosis.

Macrophages are the major infiltrating immune cells in atherosclerosis and contribute to the development of atherosclerosis by engulf modified lipoproteins via scavenger receptors and promoting foam cell formation and inflammation. Our study found that Hh signaling pathway activation facilitates macrophage-derived foam cell formation by blocking cholesterol efflux without changing oxLDL phagocytosis. Cholesterol efflux in foam cells originating from macrophages is beneficial to the reduction of lipid droplets consisting of cholesterol ester (CE). There are two main ways to clear CE in foam cells. A portion of cellular CE is hydrolyzed by neutral cholesterol esterase in the cytoplasm into free cholesterol, which effused via ABCA1, ABCG1 and aqueous diffusion. The other route of CE clearance is that CE enclosed in lipid droplets is transported into lysosomes by autophagosome and then hydrolyzed into free cholesterol by acid lipase, and those free cholesterol derived from autophagolysosomes are available for efflux via ABCA1. Autophagy, of which activation accelerates lipid metabolism and alleviates inflammatory injury, is inhibited in atherosclerosis. Activation of Hh signaling pathway was found to impair the autophagy in cancer cells [39], endometrial stromal cells [40], uroepithelial cells [41] and hepatic stellate cells [42]. Sahana Holla et al. demonstrated that Mir31 inhibits autophagy via activating Hh signaling pathway in macrophages [43]. In our study, we proved that inhibition of Hh signaling by vismodegib could promote autophagy in macrophages by usage of two autophagy inhibitors LY294002 and CQ administration. Based on that, we conjectured that the vismodegib stimulated cholesterol efflux by accelerating CE hydrolysis via activation of autophagy. We found vismodegib treatment promoted cholesterol efflux and reduced foam cell formation. The inhibition of autophagy could reverse the cholesterol efflux. Our data show that vismodegib enhanced cholesterol efflux and decreased macrophage-derived foam cell by modulating autophagy.

Our study clearly shows a role of Hh signaling inhibition in anti-atherosclerosis with reduction of plaque area and lipid content in vismodegib treatment apoE^-/-^ mice. This reduction was not dependent on changes in serum-lipid or body weight. We also prove that inhibition of Hh signaling promotes cholesterol efflux and reduces macrophage-derived foam cell formation. Previously, Dimitra Aravani et al. proved that HHIPL1 with hedgehog signaling promotion in VSMCs, contributed to pro-atherogenesis [19]. On the contrary, Beckers et al. found that using of monoclonal antibody inhibiting three Hh family proteins brought about larger lesion area in advanced atherosclerosis [20]. The discrepancy among the results of these studies might reflect the various effects of Hh signaling in different stages of atherosclerosis. Whatever, it reminds us that the involvement of Hh signaling in atherosclerosis has been affirmed and further investigations on the role of Hh signaling in the pathogenesis of atherosclerosis are required.

**Fig. 7:**
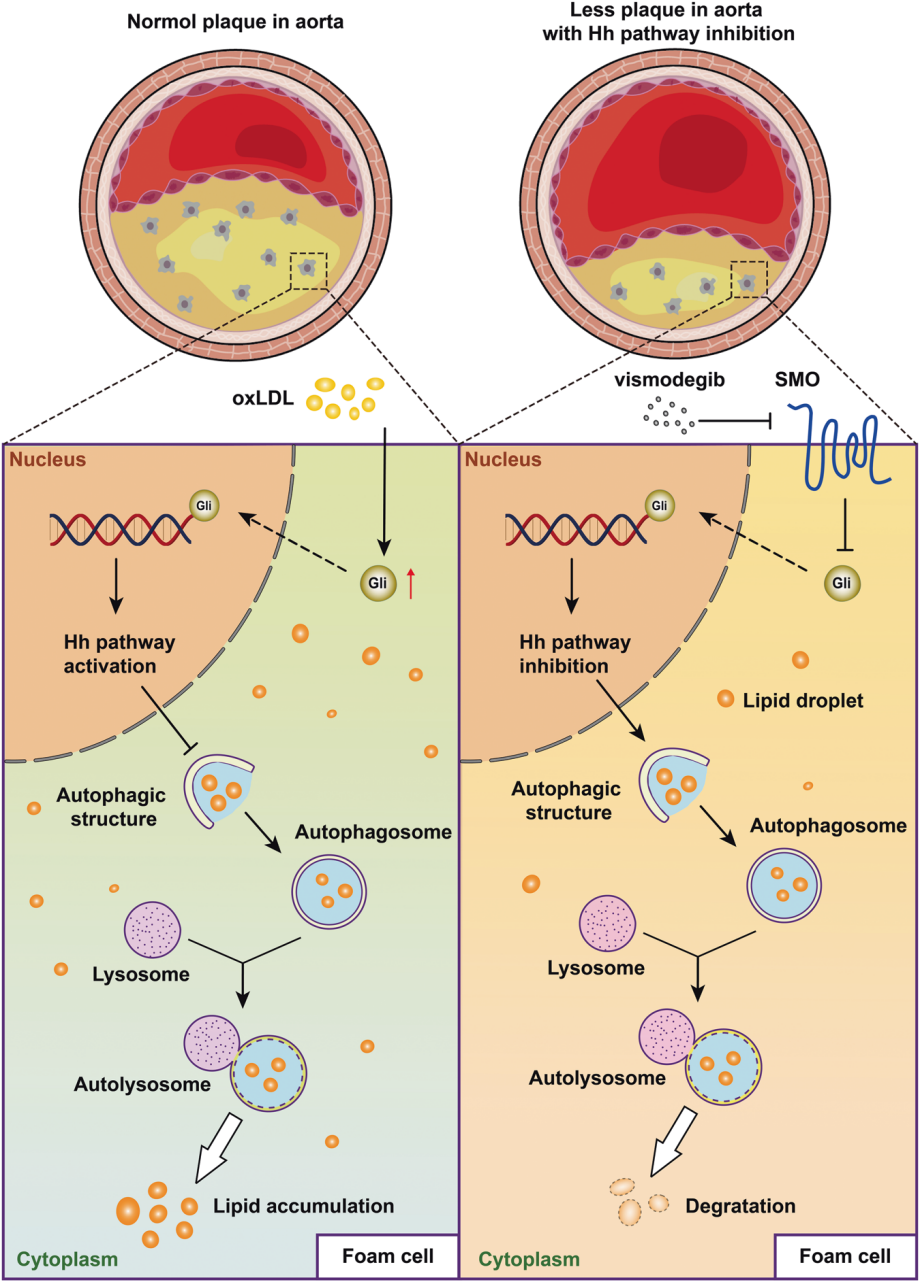
Schematic diagram of how Hh signaling activation promoted atherosclerosis. In brief, Hh signaling pathway was activated in macrophages-derived foam cell which inhibited lipophagy and caused lipid deposition. Moreover, vismodeigib administration, which inhibited Hh activation, increased autophagy efflux and lipophagy in macrophages-derived foam cell so that promoted cholesterol efflux and inhibited foam cell formation.

To sum up, our data revealed that the Hh signaling pathway is critical in manipulating macrophage cholesterol homeostasis in early atherosclerosis. Our study clarified that Hh signaling promoted lipophagic flux by accelerating autophagy, which, in turn, in macrophage-derived foam cell formation (Fig. 7). Furthermore, our results also indicated that vismodegib might be helpful for treatment of early atherosclerosis and other lipophagy-related diseases.

## Materials and Methods

### Reagents and antibodies

The reagents used are as follows: oxLDL (Angyubio, Shanghai China, AY-1501), vismodegib (Shyuanye, Shanghai China, S81056), Oil O Red (Shyuanye, S19039), BODIPY (GLPBIO, NJ USA, GC42959), Chloroquine (Sigma-Aldrich, C6628), NBD-cholesterol (Cayman, Czech Republic, 13220), apoA-I (Novoprotein, Shanghai China, DC511), LY294002 (Selleck, Texas USA, S1105), and Lyso-Tracker Red (Invitrogen, CA USA, L7528). Primary antibodies: anti-4HNE (abcam, CB2 0AX UK, ab48506) and anti-LC3 (Cell Signaling Technology, Boston USA, 3868). Secondary antibodies: anti-GAPDH rabbit pAb (Servicebio, GB11002), anti-beta Actin rabbit pAb (Servicebio, GB11001), and anti-rabbit Alexa Fluor 568-conjugated (Thermo Fisher Scientific, MA USA, A11011).

### Animals

ApoE^−/−^ mice were purchased from Cavens Biogle (Suzhou) Model Animal Research Co.Ltd (Suzhou, China). To induce atherosclerotic plaque, apoE-knockout mice (male, 6–8 weeks old) were fed with a high-fat diet (HFD) containing 30% fat, 3% cholesterol and 0.15% bile salt for 12 weeks. To inhibit hedgehog signaling pathway atherosclerosis, apoE^−/−^ mice were fed with HFD diet for 2 weeks and then administered vehicle or vismodegib (40 or 60 mg/kg) every 2 days by oral perfusion with HFD diet for another 6 weeks maintaining. Mice were allocated into 4 groups which contained 8–9 mice respectively randomized. The number of experimental animal in vivo was based on power calculation for the primary parameter (lesion area) with mean differences and standard deviations taken for pilot data at power 90% with an α of 0.05. This experiment was in accordance with the ethical statement that relate to all living-animal scientific procedures.

### Cell culture

J774a.1 cells were obtained from Nanjingbaike (Nanjing China). THP-1 cells and VSMC were purchased from ATCC. After cultured in dulbecco’s modified eagle medium (Gibco, NY USA, C11995500BT) with 10% fetal bovine serum (Gibco, 10270) at 37 °C in a humidified atmosphere with 5% CO_2_, J774a.1 cells, PMA stimulated THP-1 cells and VSMCs were incubated with oxLDL for 24 h to induce the formation of foam cells. All cell lines were kept mycoplasma-free which checked every 3 months.

### Western blot analysis

Cell were incubated with RIPA lysis buffer which used to extract total protein for 20 min at 0 ~ 4 °C. Cell lysates were collected into tubes and separated by centrifuge at 12,000 rpm, and protein concentrations were measured by BCA for western blot analysis. LC3 or β-actin rabbit antibodies were incubated with PVDF membranes overnight, and then secondary antibodies were incubated for 1 h at room temperature. The proteins were detected by chemiluminescence. The gray value of each lane was measured by Fiji (*n* = 4).

### Atherosclerotic analysis

Mice were sacrificed after 8 weeks of HFD. The aortas were dissected and fixed in 4% paraformaldehyde (Servicebio, G1101), and then embedding with OCT. The cross-sectional slices (5 μm) of aorta were cut from the beginning of aortic valve. To examine lipid content of atherosclerotic plaque, frozen sections of aortic sinus were incubated with Oil O red for 2 h at room temperature. Then slices were re-stained in haematoxylin for 90 s to reveal nuclei. Sections were observed with an optical microscope. Oil O red positive area and lesion area were calculated by Fiji software. Mean value of 5 sections (each 50 μm apart and spanning 250 μm) was calculated as the final results for analysis of each mouse (*n* = 8–9).

### Immunofluorescence analysis of aortic sinus

Frozen sections were incubated with GLI1 rabbit antibody at 4 °C overnight after 0.5% Triton treatment for 8 min. Slices were incubated with secondary antibodies and nuclei dye Hoechst 33342 at room temperature for 1 h. Staining was observed via confocal microscope (ZEISS, LSM710) and processed by Fiji software (*n* = 8–9).

### Oil O red-staining of cells

Foam cell were fixed in 4% paraformaldehyde at 4 °C overnight and then stained with Oil O red at room temperature for 15 min. Before observation by an optical microscope, cells were washed once with 60% isopropanol and three times with PBS. For quantification of ORO staining, isopropanol was used to extract ORO dye in cells and OD value of isopropanol was measured at 510 nm (*n* = 3).

### Immunofluorescence analysis of foam cells

After being fixed by 4% paraformaldehyde, cells were perforated by 0.5% Triton and incubated with secondary antibodies and Hoechst 33342 for 1 h at room temperature. Then, cells were washed three times with PBS prior to observation through a confocal microscopy. In BODIPY and Lyso-Tracker staining, perforation was needless and dyes were added to fix cells directly at 37 °C for 15 min. Mean fluorescent intensity of each slice were analyzed by Fiji software (n = 4).

### TC and TG content

The TC and TG levels of foam cells were measured by the TC (Nanjing Jiancheng, A111-1-1) and TG assay kit (Nanjing Jiancheng, A110-1-1). Intracellular cholesterol and triglyceride levels of different samples were normalized to the individual protein concentration. All experimental operations were according to the protocols of manufacture (*n* = 3).

### Cholesterol efflux assay

Macrophages were stimulated by NBD-cholesterol for 8 h in DMEM with 1% fetal bovine serum. After 4 h of equilibrium, cholesterol-loaded macrophages were incubated with vismodegib for 2 h. ApoA-I was added to the system for an hour, and then the medium was collected. Cells were lysed with 0.5% PBST at 37 °C for 10 min. BioTek Multi-Mode Microplate Reader was used to measure the fluorescence intensity of cell lysates and corresponding medium (*n* = 3). Cholesterol efflux rate was calculated according to the following formula: cholesterol efflux (%) = medium fluorescence intensity/(medium fluorescence intensity + cell lysate fluorescence intensity) × 100.

### Analysis of NBD-cholesterol uptake

Macrophages stimulated with NBD-cholesterol for 8 h were analyzed for NBD-cholesterol uptake by measuring the mean fluorescence intensity by flow cytometry (*n* = 3).

### Transmission electron microscopy

Cells were fixed in 2.5% glutaraldehyde and prepared according to previous recommendations [44]. Then, the slices of cells were commercially detected by transmission electron microscope (Servicebio, Wuhan, China).

### Cholesterol synthesis content quantification

Following 24 h of stimulation of J774a.1 cells with vismodegib and DMSO, respectively, centrifuged cells were collected and total protein was determined by BCA. Cells were hydrolyzed in 1 M NaOH in methanol and subsequently extracted using cyclohexane. Under nitrogen protection, the solution obtained from the extraction was rotary evaporated to dryness and then dissolved by adding n-decane. GC/MS detection of the content of the three cholesterol precursors was performed on a 6890 N Network GC system (AgilentTechnologies) using a DB-XLB (30 m length x 0.25 mm internal diameter, 0.25 um film) column (Agilent Technologies, Waldbronn, Germany). lanosterol, lathosterol and desmosterol standards were purchased from MCE. Individual data were finally normalized to total protein concentration.

### Statistical analysis

All experiments were performed with at least three replicates. Data were analyzed in randomized and blinded Results of this experiment were presented as mean ± standard error of mean (SEM). Shapiro–Wilk test was used to check the normality and group variance in all statistical data. The two-sided student’s *t*-test (for normal distribution data) and Mann–Whitney test (for not normal distribution data) and Bonferroni’s post-test were performed using Prism 9 software, and differences with *p* < 0.05 were considered statistically significant. Based on preliminary experiments, sample sizes were chosen to ensure a power of 80% and an alpha level of 5%. No data or animals were excluded from the analyses.

### Reporting summary

Further information on research design is available in the Nature Research Reporting Summary linked to this article.

### Supplementary information

supplementary figure legend

Figure S1

Figure S2

Figure S3

Figure S4

Reporting Summary

original data files

## Data Availability

All data generated or analyzed during this study are available from the corresponding author on reasonable request. No applicable resources were generated during the current study.
