# Supplementary material for: Inhibition of Hedgehog signaling ameliorates foam cell formation by promoting autophagy in early atherosclerosis
Source: Cell Death Dis. 2023 Nov 14;14(11):740. doi: 10.1038/s41419-023-06270-5 (PMC10646116; doi:10.1038/s41419-023-06270-5)
Supplement: Supplementary file 1 — supplementary figure legend [file 41419_2023_6270_MOESM1_ESM.docx]

**Figure S1.** **Hh signaling was activated in VSMC-derived foam cells.** (A) Relative expression of Gli1 in VSMCs treated with oxLDL at different time points (n=3). (B) Representative images of ORO-stained VSMCs were stimulated with vehicle control, Hh signaling inhibitor (vismodegib), oxLDL, or vismodegib and oxLDL for 24 hours. (C) The ORO OD values were measured at 510 nm of each group in (B) (n=3). (D to E) Total cholesterol (D) or Triglyceride (E) levels in macrophages described in (B) (n=3). * *p*<0.05, ** *p*<0.01, *** *p*<0.001

**Figure S2. Cell viability and Gli1 expression level under different concentration of vismodegib treatment.** (A) Cell viability of J774a.1 cells with vismodegib treatment in different concentration (n=3). (B) Relative Gli1 expression of J774a.1 cells with vismodegib treatment in different concentration (n=3). * *p*<0.05, ** *p*<0.01, *** *p*<0.001.

**Figure S3. Vismodegib treatment had no effect on cholesterol synthesis.** Content of total cholesterol and cholesterol precursors (lanosterol, lathosterol and desmosterol) which measured by GC/MS (n=3). * *p*<0.05, ** *p*<0.01, *** *p*<0.001

**Figure S4.** **Negative control of immunofluorescent staining.** (A) Negative control of LC3 II immunofluorescent staining. (B) Negative control of 4-HNE immunofluorescent staining.
