# Supplementary figures and images for: Inhibition of Hedgehog signaling ameliorates foam cell formation by promoting autophagy in early atherosclerosis
Source: Cell Death Dis. 2023 Nov 14;14(11):740. doi: 10.1038/s41419-023-06270-5 (PMC10646116; doi:10.1038/s41419-023-06270-5)

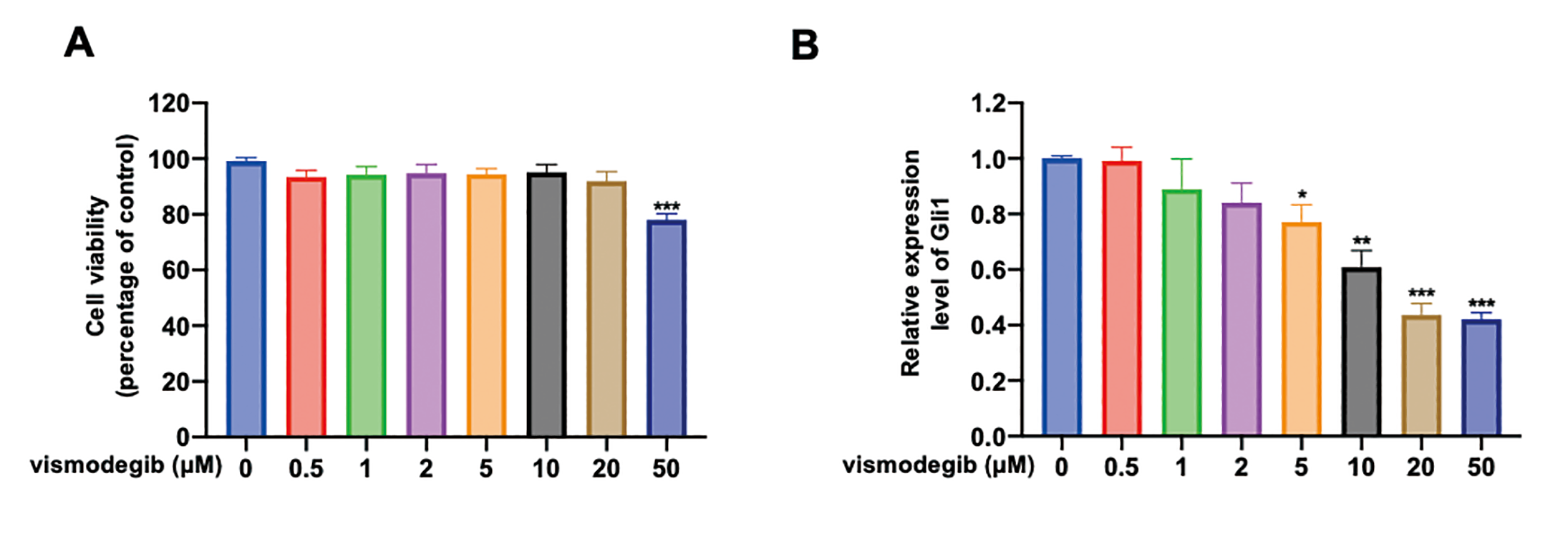

Supplement: Supplementary file 3 — Figure S2 [file 41419_2023_6270_MOESM3_ESM.png]

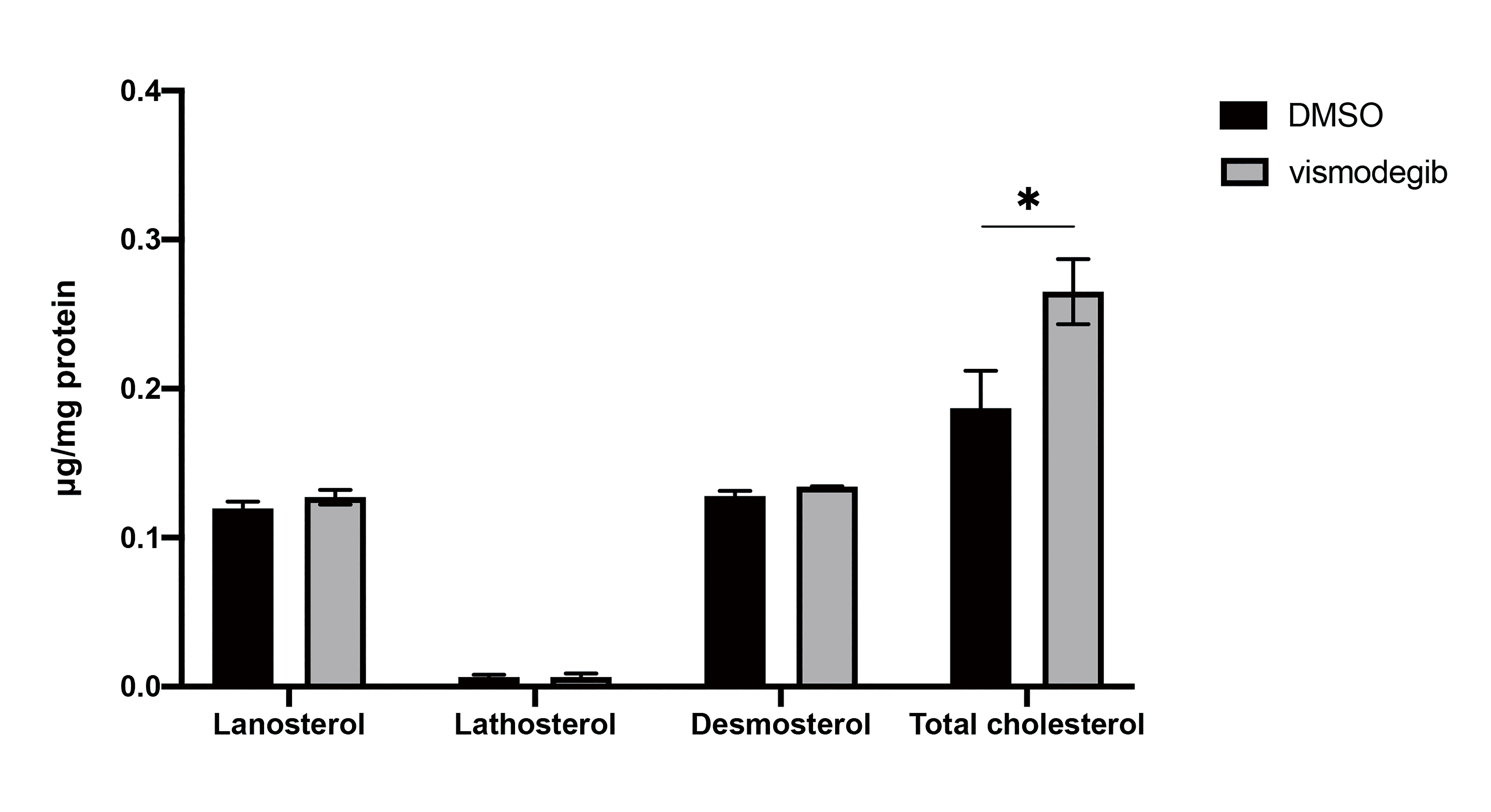

Supplement: Supplementary file 4 — Figure S3 [file 41419_2023_6270_MOESM4_ESM.png]

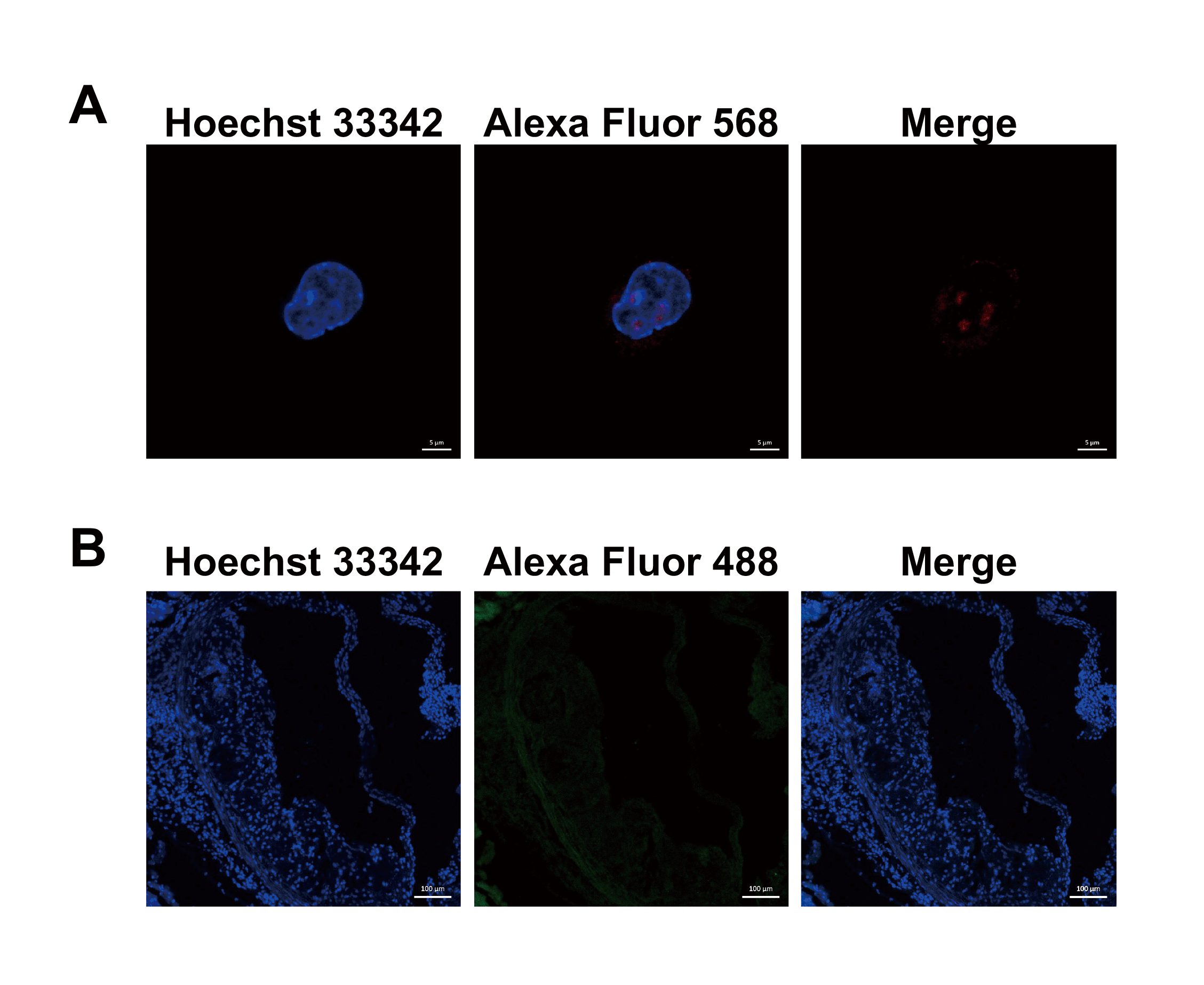

Supplement: Supplementary file 5 — Figure S4 [file 41419_2023_6270_MOESM5_ESM.png]

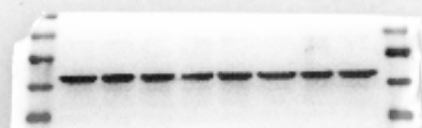

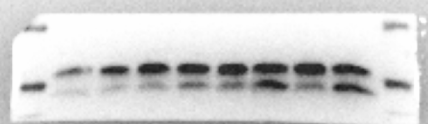

Supplement: Supplementary file 7 — original data files [file 41419_2023_6270_MOESM7_ESM.pdf]
